# Mitochondrial metabolic remodeling and multi-omics profiling identify plasma biomarkers of myocardial infarction

**DOI:** 10.1016/j.jmccpl.2025.100827

**Published:** 2025-11-13

**Authors:** Selvam Paramasivan, Mitchell C. Lock, Roberto A. Barrero, Paul C. Mills, Janna L. Morrison, Pawel Sadowski

**Affiliations:** a Florey Institute of Neuroscience and Mental Health, University of Melbourne, Melbourne, VIC, Australia; b School of Veterinary Science, The University of Queensland, Gatton, QLD, Australia; c Central Analytical Research Facility, Queensland University of Technology, Brisbane, QLD, Australia; d Clinical and Health Sciences, University of South Australia, Adelaide, SA, Australia; e eResearch, Research Infrastructure, Academic Division, Queensland University of Technology, Brisbane, QLD, Australia

**Keywords:** Myocardial infarct, Mitochondria, Upstream regulators, Proteomics, Multi-omics, Plasma biomarkers

## Abstract

Mitochondrial dysfunction is a hallmark of myocardial infarction (MI), yet the molecular mechanisms linking metabolic reprogramming in the ischemic myocardium to systemic biomarker signatures remain incompletely understood. In this study, we employed a data-independent acquisition mass spectrometry (SWATH-MS) strategy integrating multi-omics analysis with upstream regulatory network analysis to investigate mitochondrial energy pathway alterations in a preclinical ovine model of MI. Proteomic profiling of infarcted myocardium revealed a pronounced shift from oxidative phosphorylation to glycolysis, accompanied by coordinated suppression of mitochondrial fatty acid β-oxidation enzymes. This metabolic reprogramming was strongly associated with four upstream master regulators, most notably predicted inhibition and significant transcriptional downregulation of *PPARGC1A*, a key coactivator of mitochondrial biogenesis and oxidative metabolism, indicating disrupted mitochondrial energy homeostasis and impaired adaptive responses in ischemic cardiomyocytes. Parallel plasma proteomic analysis identified a distinct panel of differentially expressed proteins enriched in pathways related to carbon metabolism, amino acid biosynthesis, and cardiac muscle contraction. Notably, mitochondrial metabolic enzymes such as *SUCLG1*, *MDH2*, *HADHA*, and *HADHB* were significantly downregulated at both the transcript and protein levels in cardiac tissue, while their protein abundance was markedly increased in plasma post-MI, highlighting their potential as circulating biomarkers of mitochondrial dysfunction. These findings provide mechanistic insight into the energy metabolic remodeling that occurs during myocardial ischemic injury and establish a systems-level framework for linking tissue-specific mitochondrial alterations with accessible plasma biomarkers. This study supports the translational potential of targeting mitochondrial pathways for diagnostic and therapeutic strategies in ischemic heart disease.

## Highlights

• Mitochondrial metabolic remodeling contributes to cardiomyocyte death post-MI.
• TCA cycle, OXPHOS, and fatty acid oxidation enzymes are downregulated.
• Inhibited PPARGC1A disrupts mitochondrial energy homeostasis in ischemic heart.
• SUCLG1, MDH2, HADHA, and HADHB are altered in tissue and plasma post-MI.

## Graphical abstract

**Figure:**
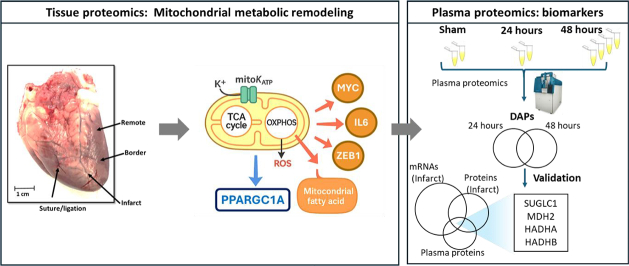

## 1 Introduction

Cardiovascular disease (CVD) is the leading global cause of death accounting for approximately 25 % of fatalities and thus poses a significant public health burden [[1], [2], [3]]. Myocardial infarction (MI), a common and severe manifestation of CVD, exerts profound effects on myocardial tissue, particularly on cardiomyocytes, the primary contractile units of the heart. During MI, complex interactions between intra- and extracellular proteins drive distinctive remodeling within the microvascular environment, disrupting oxygen and nutrient delivery to affected myocardial regions and resulting in tissue damage or necrosis [4]. Despite the challenges in characterizing the infarcted tissue, elucidating the underlying molecular mechanisms provides a foundation for developing targeted interventions to enhance endogenous repair and tissue recovery. Critical to these efforts is the precise characterization of protein network patterns and dynamics that define the molecular signatures of both healthy and infarcted tissues. Such insights are pivotal for advancing our understanding of adult cardiomyocyte responses to pathogenic stimuli and for enabling early detection and intervention in the disease process.

The intricate interplay between mitochondrial metabolism and the regulation of gene expression encompassing transcription factors, microRNAs, and proteins represents a fundamental aspect of cellular signaling networks [[5], [6], [7], [8]]. This dynamic regulatory system orchestrates a balance between metabolic and gene expression processes, critically influencing cellular function and adaptability. In the context of MI, this interplay contributes to the structural and functional remodeling of cardiomyocytes, where metabolic perturbations disrupt mitochondrial adenosine triphosphate (ATP) production, impairing the myocardium's contractile efficiency [9,10]. Although accumulating evidence implicates alterations in mRNA, microRNAs, and proteins in the progression of myocardial infarction, the integrated contribution of these regulatory networks in linking mitochondrial dysfunction within infarcted and peri-infarct myocardial regions to the resulting pathological proteomic phenotype and systemic biomarker signatures remains poorly defined.

This study aimed to investigate mitochondrial metabolic remodeling in infarcted and adjacent myocardial zones using a preclinical ovine model. By integrating SWATH-based data-independent acquisition (DIA) mass spectrometry with multi-omics analysis and upstream regulatory network modeling, we sought to characterize proteomic alterations within the infarcted myocardium, with a specific focus on dysregulated mitochondrial energy pathways. Emphasis was placed on elucidating the regulatory role of PPARGC1A inhibition in driving the suppression of oxidative metabolism. In parallel, plasma proteomic profiling was performed to identify differentially expressed proteins reflective of myocardial mitochondrial dysfunction. Together, these approaches aimed to establish a systems-level understanding linking tissue-specific metabolic disruptions to circulating biomarker signatures, thereby informing the development of targeted diagnostic and therapeutic strategies for ischemic heart disease.

## 2 Methods

All animal procedures were approved by the South Australian Health and Medical Research Institute (SAHMRI) Animal Ethics Committee (SAM046) and conducted in accordance with the Australian Code for the Care and Use of Animals for Scientific Purposes. The study followed ARRIVE guidelines [11] and the 3Rs principles [12].

### 2.1 Ovine sample collection

Eight adolescent (∼6-month-old) Merino sheep were housed under controlled environmental conditions (20–22 °C, 12:12 h light-dark cycle) with ad libitum access to food and water. Under general anaesthesia (IV diazepam 0.3 mg/kg and ketamine 7 mg/kg; maintained with 1–2 % isoflurane), thoracotomy was performed and a jugular vein catheter inserted [13]. Sheep were randomly assigned to Sham (*n* = 4) or left anterior descending (LAD) coronary artery ligation (n = 4) groups [[13], [14], [15], [16]]. In the infarction group, a silk suture was placed around the second diagonal of the LAD coronary artery, and infarction was confirmed by regional blanching. Antibiotic prophylaxis (Procaine penicillin 154 mg, benzathine penicillin 393 mg, dihydrostreptomycin 500 mg) and analgesia (xylazil 20 μg/kg) were administered during and post-surgery.

Blood samples were collected at 24 and 48 h post-surgery. On day 3, sheep were euthanized with sodium pentobarbitone (8 g IV). Hearts were excised, photographed and then briefly cannulated to prevent clotting and arrest the heart in diastole. Heparin sulphate (500 IU) was diluted in a Ca^2+^ free solution containing (in mmol/L) NaCl, 134; glucose, 11; HEPES, KCl, 4; MgSO4, 1.2; NaH2PO4, 1.2.

**Fig. 1:**
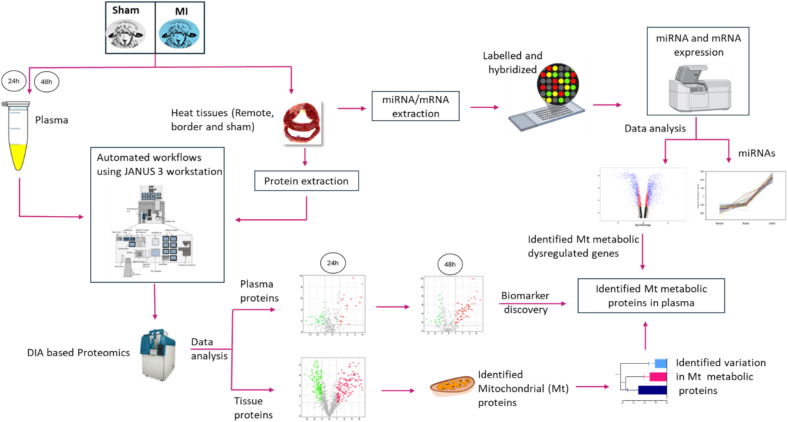
Overview of the experimental workflows employed for proteomic and transcriptomic analyses. Heart tissue samples, including infarct, border, and remote regions, were collected from sham operated and MI groups. RNA was extracted from each tissue region, labeled with cyanine 3 dye, hybridized to a custom ovine gene microarray and scanned with an Agilent G2505B 2 dye scanner. Plasma samples were obtained at 24 and 48 hour post-surgery. Proteomic sample preparation was performed using an automated workflow on the GANUS-3 workstation. Both tissue and plasma proteomes were analysed using DIA-based mass spectrometry to enable biomarker discovery.

The whole heart was then sectioned (∼0.5 cm per section), and snap frozen for molecular analysis, processed for histology (submerged in 4 % paraformaldehyde) or stained with TTC for visualization of the infarct area. Triphenyltetrazolium powder was diluted in phosphate buffer solution (pH 7.4) as 1 % weight/volume (1 g/100 mL). Tissue slices were stained at 37 °C for 10 min, followed by fixation in 10 % formalin at room temperature. Plasma and tissue samples were stored at −80 °C until proteomic and transcriptomic analyses. Fig. 1 illustrates the workflow outlining the sample preparation and analytical procedures employed in this study.

### 2.2 RNA extraction and gene microarray procedure

Total RNA was extracted from frozen heart tissue for each fetus and adolescent sheep using QIAzol Lysis Reagent solution and QIAgen miRNeasy purification columns, as per manufacturer guidelines (Qiagen, Germany) as previously described [13,[15], [16], [17]]. Total RNA was quantified by spectrophotometric measurements at 260 and 280 nm in a NanoDrop Lite Spectrophotometer (ND-1000, ThermoFisher, Wilmington DE) and its integrity was measured with an Agilent Bioanalyzer, 2100 model (RIN 7.6–9.5). One μg RNA per sample was labeled with Cy3 using the Agilent Quick Amp Kit and hybridized to Agilent 8 × 15 k ovine microarray slides (GPL14112 platform) at 65 °C for 17 h. Slides were scanned (Agilent G2505B), and raw data submitted to NCBI GEO (GSE144509) [13].

Data were imported into R using the limma package [18], background-corrected, and quantile normalized. Low-expression probes (*n* = 7631) were filtered out. Probe redundancy was resolved via Weighted Gene Co-expression Network Analysis (WGCNA) [19], yielding 5696 unique genes. Differential expression between groups (Infarct vs. Sham, Infarct vs. Remote, Border vs. Remote, Infarct vs. Border) was determined using moderated *t*-tests with Benjamini–Hochberg correction (adjusted *p* < 0.05) and log₂ fold change ≥±1.

### 2.3 miRNA microarray

miRNA profiling was performed using a custom array (LC Sciences, USA) containing 3098 miRNA probes (including ovine and conserved mammalian sequences) and 56 controls, with multiple technical replicates [20]. RNA from the same tissue samples used in mRNA analysis was used. Redundancy was expected due to conserved multispecies sequences. Three biological replicates per treatment group were analysed. One sample did not meet the required RNA quality threshold (RNA Integrity Number, RIN > 8.0) and was excluded; therefore, *n* = 3 samples were included in the final analysis for this experiment.

### 2.4 Proteomics sample preparation

Protein extraction and digestion followed our established automated workflow [17]. Heart tissues (∼3 mg) were homogenized in lysis buffer (1 % sodium deoxycholate, 100 mM DTT in 100 mM Tris-HCl, pH 8.5) using a TissueLyser II (Qiagen) and tungsten beads. Plasma (5 μL) was lysed in 20 μL buffer [21]. Protein concentrations were measured via BCA assay (Pierce). Samples were digested using a modified FASP protocol on a JANUS G3 workstation with 30 kDa MWCO filter plates. Peptides were eluted, desalted, and fractionated (SCX and high-pH reverse-phase) using StagePlates in 96-well format. Dried peptides were reconstituted in 2 % ACN/0.1 % FA with iRT standards and quantified using a colorimetric peptide assay (Thermo Fisher Scientific).

### 2.5 LC-MS/MS proteomics analysis of sheep heart and plasma samples

Peptide samples were analysed using a SCIEX TripleTOF 6600 system with microflow DuoSpray source and Ekspert nanoLC [17,21]. In DDA mode, the 40 most intense precursor ions (charge 2–5) were selected (250 ms accumulation, *m*/*z* 400–1250), fragmented, and dynamically excluded for 18 s. In DIA (SWATH) mode, 60 variable windows (*m/z* 399.5–999.7) with 1 *m/z* overlap were employed. TOF-MS scans (*m/z* 300–1500, 50 ms) and SWATH-MS scans (*m/z* 100–1800, 50 ms) resulted in ∼3.1 s total cycle time. Collision energy was optimized for doubly charged peptides, and declustering potentials were tuned accordingly.

### 2.6 DIA data processing

Sheep heart DIA data were analysed using Skyline [17]. The spectral library was recalibrated using iRT peptides. Peptides with ≥1 proteotypic sequence and ≥5 transitions were quantified (0.05 *m/z* fragment tolerance). DIA signal identification was restricted to a ±2 min RT window. Peptide scoring used the mProphet model (q < 0.01). Data normalization used median equalization. MSstats was employed to confirm technical reproducibility and remove bias. Plasma DIA data were processed using Spectronaut (version 17, Biognosys) in DirectDIA mode. DIA data were processed using Spectronaut's Pulsar search engine to generate a spectral library, which was subsequently used for targeted analysis and accurate peptide quantification. Protein and precursor identifications were filtered using a normal distribution estimator with a q-value (FDR) cutoff of <0.01. For quantification, interference correction was applied, and MS2 ion peak areas of quantified peptides were summed to calculate protein abundances, using the area under the curve within integration boundaries. Precursor quantities were normalized using the built-in global median normalization function in Spectronaut, enabling comprehensive and consistent proteomic profiling.

Functional enrichment and pathway analyses were performed using Ingenuity Pathway Analysis (IPA) and ClueGO [22]. For the upstream regulator analysis, a *Z*-score greater than 2.0 was defined as the threshold for significant activation, whereas a Z-score less than −2.0 was defined as the threshold for significant inhibition. An overlap *p*-value of <0.05 was applied as an additional criterion for statistical significance.

### 2.7 miRNA interaction network analysis

To identify potential post-transcriptional modulators of proteomic biomarkers in adolescent sheep Infarct tissues, we hypothesised that downregulated miRNAs in the same tissues may account in part for the upregulation of protein biomarkers [16]. Overall, 176 miRNA accessions including 85 cattle, 49 human and 42 mouse mature miRNA sequences were selected. These miRNAs represent a set of 109 non-redundant genes encoded in 73 miRNA precursors. Predicted targets for each individual miRNA accession were identified in Target Scan release 7.2 [23]. Initially, predicted vertebrate miRNA targets for 80 out of 176 miRNA accessions were used to map to identify protein markers. Next, to identify corresponding orthologous sheep miRNA target genes, a reciprocal best hit approach [24] was used to find orthologous matches in the sheep transcriptome (Oar rambouillet v1.0). Prior miRNA target prediction sheep genes with a 3’-UTRs of at least 100 bases in length were selected. PITA [25] was then used to predict putative miRNA binding sites in the 3’ UTRs of sheep genes. Significantly differentially abundant proteins (DAPs), genes (DEGs) and miRNAs (DEMIs) were used for quantitative analysis.

### 2.8 Histological staining and image capture

Fixed tissues were embedded into paraffin blocks and 5 μm thickness sections were cut on a Leica HistoCore manual microtome (Leica Biosystems, Germany). Sections were stained in Hematoxylin and Eosin by a service provider (University of Adelaide Histology Services) and digitised using a NanoZoomer XR slide scanner (Hamamatsu, Japan) at 40× magnification.

### 2.9 Statistical analysis

Experimental data are presented as mean ± standard error of the mean (SEM). Comparisons among different myocardial regions were conducted using one-way analysis of variance (ANOVA), followed by Tukey's post hoc multiple comparisons test. All statistical analyses were performed using GraphPad Prism version 10. A *p*-value of less than 0.05 was considered statistically significant. LF means log fold change.

## 3 Results

### 3.1 Characterization of cardiac tissue in infarct, border, and remote zones in adolescent sheep following MI

**Fig. 2:**
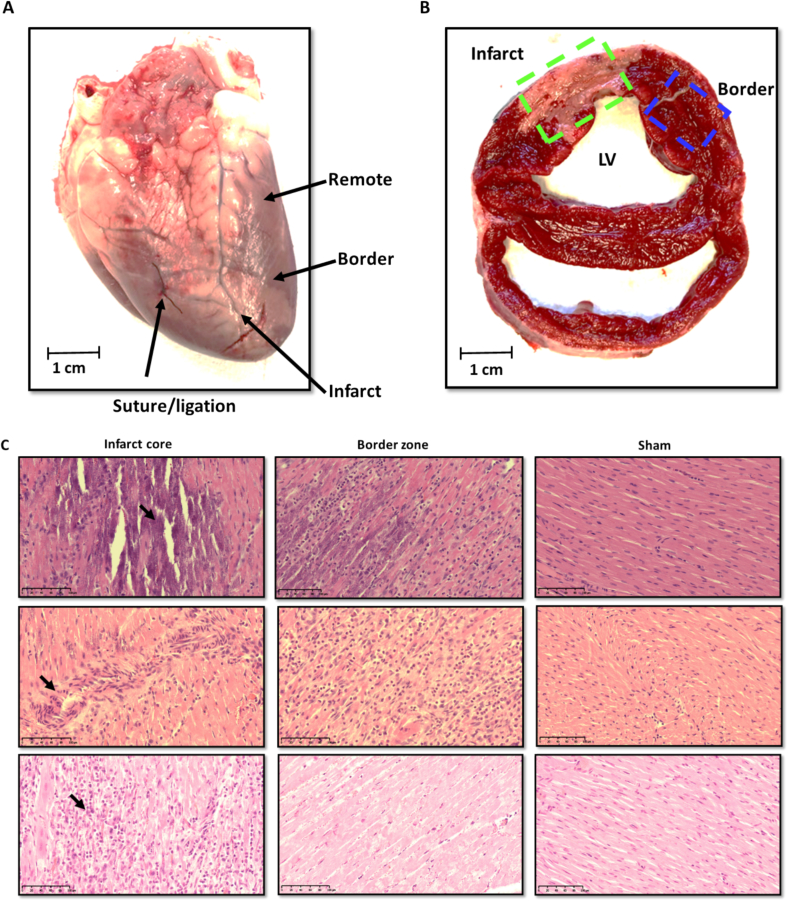
The macroscopic characteristics of an infarcted sheep heart, corresponding cardiac tissue stained with 2,3,5-triphenyltetrazolium chloride (TTC) and histological sections stained with hematoxylin and eosin (H&E). (A) Sheep heart three days post-MI, with arrows indicating the ligation site and the sources of samples from different regions (infarct, border, and remote). (B) Cross-section of the left ventricle with sampled regions. The transmural extent of the infarction is evident by a paler hue, with tissue samples systematically gathered from distinct zones: the infarct zone (indicated by the green dashed line), and the border zone (blue dashed line). The remote sample was collected from a distant part of the ventricular region as indicated in (A). (C) Technical replicates of hematoxylin & eosin-stained micrographs of sheep myocardial tissue from infarct, border (as indicated in (B)) and sham regions captured at 20× magnification (scale bar = 100 μm). Black arrows indicate noticeable myocardial necrosis is observed in the infarct core on day 3 post MI, and less visible in the salvageable border zone. (For interpretation of the references to colour in this figure legend, the reader is referred to the web version of this article.)

The explanted whole heart from a sheep subjected to ligation of the LAD coronary artery is shown in Fig. 2A. Cross-sectional analysis revealed a substantial area of transmural MI in the anteroseptal region, identified using TTC staining. The peri-infarct area, termed the border zone, is depicted in Fig. 2B. Additionally, a remote myocardial sample was collected from a region of the ventricle distant from the infarct site (Fig. 2A), serving as a control, similar to that of a sham-operated animal.

Histological examination of H&E-stained tissue sections, three days post-MI, revealed several distinctive pathological features (Fig. 2C). Necrotic cardiomyocytes demonstrated increased eosinophilia, appearing bright pink to red, as indicated by the black arrows denoting enhanced eosin dye uptake. This increased eosinophilia reflects cytoplasmic protein denaturation and a loss of cellular detail. The nuclei of these necrotic cells showed condensation and fragmentation, resulting in the absence of visible nuclei within the necrotic regions. Surrounding tissue displayed signs of hemorrhage, attributed to injury and the subsequent inflammatory response. In the salvageable border zone, a heterogeneous cell population was observed, with nuclei showing shrinkage and fragmentation, albeit to a lesser extent than in the fully necrotic areas. This border zone also demonstrated disorganized and irregular cellular arrangements, inflammatory infiltration, and early signs of fibrosis.

### 3.2 Comparison of infarct, border, and remote proteomes reveals significant alterations in mitochondrial protein composition

**Fig. 3:**
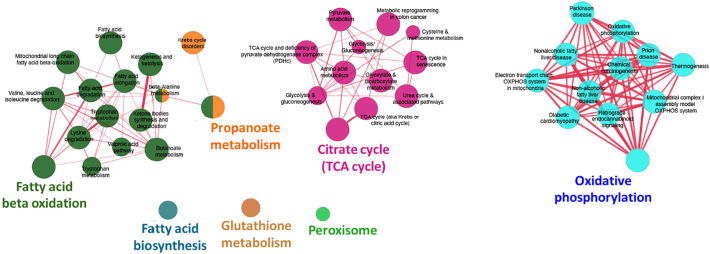
Functional network analysis of 98 downregulated mitochondrial proteins identified in the infarct region relative to both the border and remote regions (from a total of 102 significantly dysregulated proteins; see Supplementary File 1). Networks were constructed from KEGG (Kyoto Encyclopedia of Genes and Genomes)/Reactome pathways as well as Gene Ontology (GO) terms. Functionally related terms were clustered according to shared protein composition using the kappa score. Each cluster is represented by a distinct colour, with only the most significant term within each cluster displayed as the representative label. The size of each node reflects the statistical significance of the corresponding enrichment. (For interpretation of the references to colour in this figure legend, the reader is referred to the web version of this article.) Functional network analysis of 98 downregulated mitochondrial proteins identified in the infarct region relative to both the border and remote regions (from a total of 102 significantly dysregulated proteins; see Supplementary File 1). Networks were constructed from KEGG (Kyoto Encyclopedia of Genes and Genomes)/Reactome pathways as well as Gene Ontology (GO) terms. Functionally related terms were clustered according to shared protein composition using the kappa score. Each cluster is represented by a distinct colour, with only the most significant term within each cluster displayed as the representative label. The size of each node reflects the statistical significance of the corresponding enrichment. (For interpretation of the references to colour in this figure legend, the reader is referred to the web version of this article.)

In our comprehensive proteomic analysis of sheep cardiac tissue, we quantified 1252 proteins across infarct, border, and remote regions of the heart [17]. Cross-referencing these proteins with mitochondrial proteome databases such as MitoCarta 3.0 [26] revealed that 281 proteins localized to mitochondria were consistently detected in all three regions. Among these, 102 mitochondrial proteins were found to be significantly dysregulated in the infarct region relative to both the border and remote myocardial areas (LF > 1, *p* < 0.05) (Supplementary 1). Functional enrichment analysis identified that 98 of these proteins were downregulated, primarily impacting key metabolic pathways including fatty acid metabolism, the tricarboxylic acid (TCA) cycle, and oxidative phosphorylation (Fig. 3). This region-specific dysregulation underscores the critical role of mitochondrial dysfunction in the pathology of myocardial infarction.

**Fig. 4:**
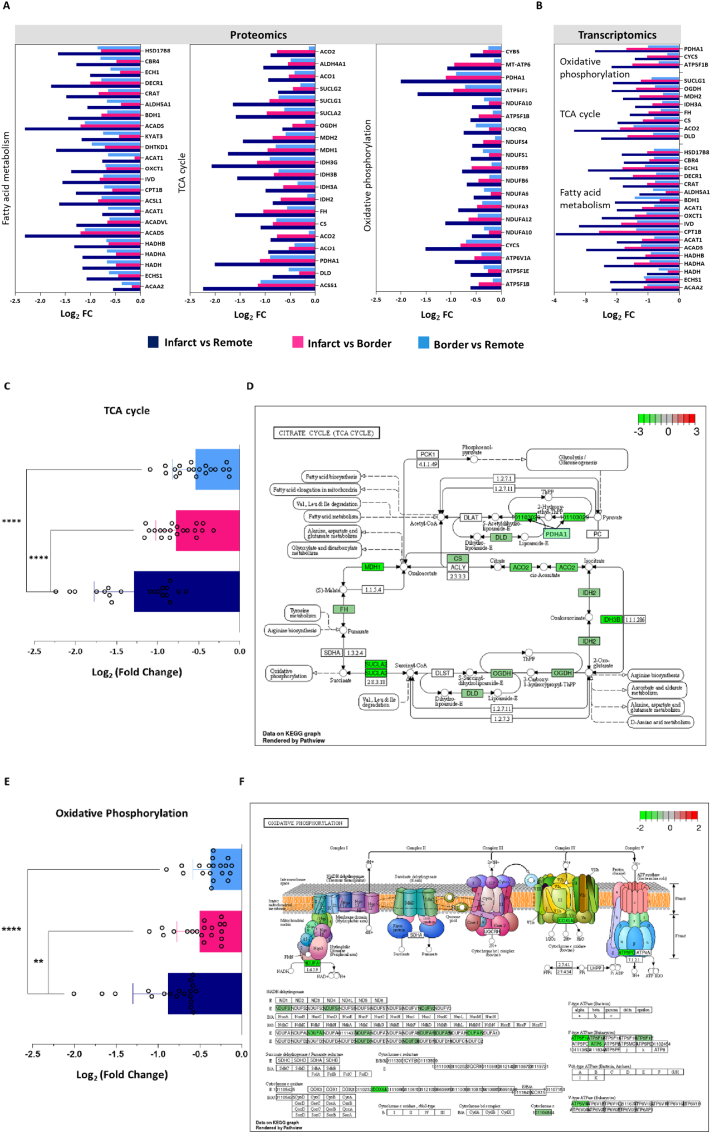
Comparison of mitochondrial protein in infarct versus remote, infarct versus border and border versus remote (A) DIA-MS analysis was used to compare a targeted set of mitochondrial proteins in infarct, border, and remote regions. Histogram bars indicate relative differences indicated based on proteomics data between the regions (infarct versus remote region (navy blue), infarct versus border region (pink) and border versus remote region (azure blue)). (B) RNA extracted from infarct, border, and remote tissues was analysed via microarrays, and the quantified mRNAs obtained through transcriptomics were compared across the three regions. (C) Analysis of variance comparing infarct versus remote regions (navy blue) and border versus remote regions (azure blue) as well as infarct versus remote regions (navy blue) and infarct versus border regions (pink) shows a significant reduction in total mitochondrial proteins associated with tricarboxylic acid cycle (ANOVA *P* *<* 0.0001, *P* *<* 0.0004) (E) Oxidative Phosphorylation (ANOVA *P* *<* 0.0001, *P* *<* 0.0003). (D) Proteomics-based pathway visualization using Pathview [27] illustrating KEGG maps of the Tricarboxylic acid cycle (F) and oxidative phosphorylation in the infarct region relative to the remote region. Downregulated proteins are indicated by green boxes. (For interpretation of the references to colour in this figure legend, the reader is referred to the web version of this article.)

Of the 98 functionally annotated mitochondrial proteins, 62 were consistently downregulated in the infarct region compared with both the border and remote regions. These dysregulated proteins were predominantly associated with key metabolic pathways, including fatty acid metabolism (*n* = 23), the tricarboxylic acid (TCA) cycle (*n* = 20), and oxidative phosphorylation (*n* = 19) (Fig. 4A). This downregulation was corroborated at the mRNA level (LF > 1, *p* < 0.05) in infarct samples (Fig. 4B), while no considerable changes in mitochondrial mRNA levels (*n* = 3; LF > 1) were observed in the remote region compared to sham controls. Notably, 59 proteins displayed dysregulation at the protein level despite stable mRNA expression (Supplementary 2), highlighting a divergence between transcriptional and translational regulation. In addition to mitochondrial dysfunction, we identified extracellular matrix (ECM) remodeling as a significant feature, with 24 proteins and 86 mRNAs showing alterations across the infarct, border, and remote regions (Supplementary 3). The infarct region exhibited the greatest ECM perturbations, consistent with increased collagen deposition observed in myocardial tissue following infarction (Fig. 2C) [13].

Further quantitative analysis demonstrated a pronounced reduction in overall abundance of mitochondrial proteins associated with the TCA cycle (*p* < 0.0001) and oxidative phosphorylation (p < 0.0001) in the infarcted region compared to the remote region. Similarly, marked and statistically significant differences were observed in these pathways when comparing the infarcted region to the border region (TCA cycle: *P* < 0.0001; oxidative phosphorylation: *P* = 0.0015; Fig. 4C and E). Proteomic pathway visualization confirmed extensive disruptions in cardiac energy metabolism, particularly within the TCA cycle and oxidative phosphorylation pathways (Fig. 4D and F). While alterations in fatty acid metabolism were also pronounced, they remained statistically significant in infarcted versus remote samples compared to the other two comparisons. These findings highlight a multifaceted impairment of mitochondrial function, underscoring their critical roles in the pathogenesis of myocardial infarction and the progression to heart failure.

### 3.3 Proteomics analysis of upstream regulators in the infarcted heart

The proteomic analysis of upstream regulators influencing alterations in the proteome of infarcted ovine hearts aimed to explore biological and molecular interactions within the identified proteins in infarcted tissues. In this study, we utilized 301 differentially abundant proteins in infarcted tissue [17] for Ingenuity Pathway Analysis (IPA). Upstream regulator analysis identified 30 genes with significant regulatory potential, determined by *Z*-scores (≤−2.0 or ≥2.0) and overlap *p*-values. Of these, 20 regulators were predicted to be activated and 10 to be inhibited (Supplementary File 4). Collectively, these upstream regulators were associated with the regulation of 134 out of 301 differentially abundant proteins, underscoring their central role in orchestrating the observed proteomic changes. Notably, the analysis revealed several transcription factors as key upstream regulators with strong activation signals, including *MLXIPL* (*Z*-score = 4.359; *P* = 7.67 × 10^−18^), *MYC* (Z-score = 2.696; *P* = 1.47 × 10^−19^), *CEBPB* (Z-score = 2.197; *P* = 1.32 × 10^−7^), *ZEB1* (Z-score = 2.178; *P* = 1.41 × 10^−6^), *STAT3* (Z-score = 2.171; *P* = 1.29 × 10^−5^), *HNF4A* (Z-score = 2.067; *P* = 3.22 × 10^−14^), *IRF2* (Z-score = 2.000; *P* = 0.00933), and *PRDM1* (Z-score = 2.433; *P* = 0.00853). Conversely, several transcriptional regulators were significantly inhibited, including *TFAP2A* (Z-score = −2.236; *P* = 0.00652), *PPARGC1A* (Z-score = −2.229; *P* = 3.76 × 10^−7^), *MYOD1* (Z-score = −2.443; *P* = 0.000561), and *ZBTB7B* (Z-score = −2.000; *P* = 2.12 × 10^−5^). These findings suggest that infarction induces complex regulatory networks involving both activation and repression of key transcriptional programs. The identification of these upstream regulators provides novel insights into the cellular processes and signaling pathways driving myocardial remodeling, and offers potential targets for therapeutic intervention in ischemic heart disease.

### 3.4 Integrative multi-omics analysis reveals regulatory networks in infarct tissues

**Fig. 5:**
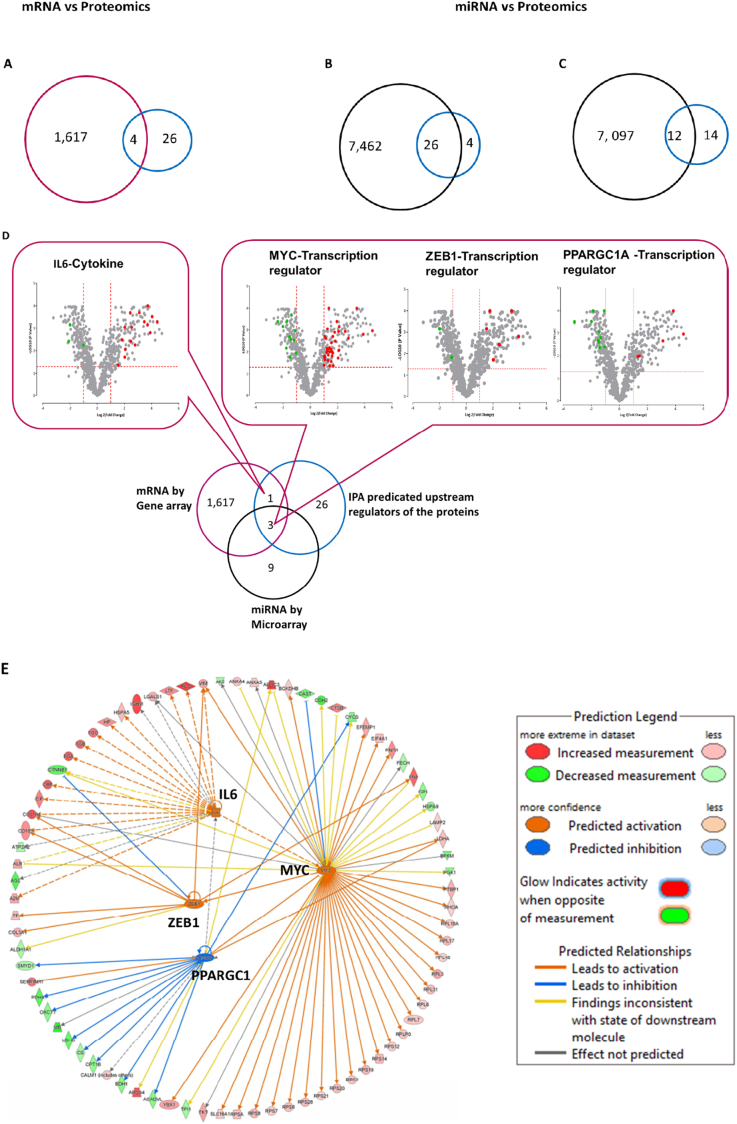
Integrative analysis of protein, mRNA and miRNA expression profiles for gene regulatory network inference (A–B). Overlap between 30 upstream regulators identified using the Upstream Regulator Analysis tool of IPA from proteins that changed upon Infarction (blue) and (A) 1621 differentially expressed genes (red), (B) 7488 human miRNA gene targets (black) inferred for downregulated miRNAs in adolescent Infarct [16]. Out of 7488 miRNAs, 26 overlapped with upstream regulators. (C) Overlap between 26 genes identified (blue) in (B) and 7109 orthologous sheep genes (black) identified 12 overlapping upstream regulators with miRNA binding sites. (D) Identification of four upstream regulators (that include three transcription regulators: *PPARGC1A, ZEB1, MYC* and one cytokine: *IL6*) because of consensus between proteomics, mRNA and miRNA analyses. The Venn diagram shows an overlap between 30 upstream regulators predicted from proteomics data (blue), 1621 genes measured using microarray (red) and 12 miRNA target genes (black) identified in (C). The inserts with volcano plots highlight proteins annotated as regulated by the consensus upstream regulators in IPA. (E) A combined network map for 79 proteins regulated by the consensus upstream regulatory genes. Particularly significant *P* values and Z score values were for inhibition of *PPARGC1A* (*P* = 3.7600E−07, *Z* score = −2.229) and activation of *MYC* (*P* = 1.4700E−19, *Z* score = 2.696), *ZEB1* (*P* = 1.4100E−06, *Z* score = 2.178) and *IL6* (*P* = 1.1200E−04, *Z* score = 2.897). Noteworthy, MYC is activated by both *IL6* and *PPARGC1* and *ZEB1* is activated by MYC. (For interpretation of the references to colour in this figure legend, the reader is referred to the web version of this article.)

Next, we employed an integrative multi-omics approach to unravel the complex regulatory networks governing the molecular responses at 3 days post infarction. Transcriptional regulators predicted by IPA were systematically cross-referenced with mRNA and microRNA (miRNA) expression datasets generated from the same samples, enabling the identification of coordinated regulatory relationships across different molecular layers. Microarray profiling of 5696 genes identified 1621 differentially expressed genes, with stringent criteria (*P* < 0.05 after false discovery rate correction and fold-change >2). Four genes demonstrated overlap with upstream regulators predicted via mass spectrometry data (Fig. 5A).

To explore the relationship between differentially abundant proteins and miRNA regulation, we initially utilized TargetScan7.2 [23] along with a set of 176 downregulated miRNAs previously published by our group [16]. This approach aimed to predict 7488 evolutionarily conserved miRNA gene targets, considering conservation across cattle, mouse, and human [28,29], which were subsequently compared with the upstream regulators (Fig. 5B). Among the identified targets, 26 human orthologous genes exhibited overlap, with 19 predicted to contain one or more miRNA binding sites within their sequences. To validate the presence of miRNA regulatory sequences in sheep genes, we first identified human-sheep orthologous genes using a best reciprocal hit approach [24], yielding a total of 7109 pairs. Subsequently, the PITA tool [25] was employed to predict miRNA binding sites in the 3’UTRs of sheep genes, revealing 12 genes that overlapped with the upstream regulators of interest, including *ESRRG, FGFR1, LARP1, MYC, MYOD1, PPARGC1A, PRDM1, SAV1, STAT3, TFAP2A, TGFB1*, and *ZEB1* (Fig. 5C).

IPA regulatory network analysis identified the MYC proto-oncogene (*MYC*), zinc finger *E*-box binding homeobox 1 (*ZEB1*), peroxisome proliferator-activated receptor gamma coactivator 1-alpha (*PPARGC1A*), and interleukin-6 (*IL6*) as key upstream regulators. Together, these regulators were predicted to influence the expression of 79 downstream proteins that showed significant differential abundance following myocardial infarction (Fig. 5D). Bioinformatics validation using transcriptomic data revealed significant alterations in key transcriptional regulators. Notably, *PPARGC1A* and *ZEB1* transcripts were markedly downregulated, corroborating the predicted inhibition of *PPARGC1A* and implicating its central role in impaired mitochondrial metabolism and energy homeostasis (Fig. 5D). In contrast, *MYC* mRNA expression was significantly elevated, consistent with its predicted activation, highlighting its involvement in driving metabolic reprogramming within the altered regulatory network. Additionally, *IL6* expression was significantly upregulated at the mRNA level, in line with its predicted activation, implicating this pro-inflammatory cytokine as a mediator of the post-infarction response (Fig. 5E). Concordant protein expression profiles in the infarcted myocardium further supported functional inhibition of *PPARGC1A* and activation of *MYC*, *ZEB1*, and *IL6*, indicating a coordinated regulatory shift that disrupts mitochondrial biogenesis and compromises cellular metabolic homeostasis. These findings underscore a tightly orchestrated regulatory mechanism linking transcriptional changes to proteomic remodeling during the early phases of myocardial infarction.

### 3.5 Exploring the discovery of plasma protein biomarkers in early myocardial infarction

We aimed to validate the hypothesis that cardiac proteins associated with myocardial infarction, as identified in sheep cardiac samples, could serve as informative plasma biomarkers for myocardial infarction. To achieve this, we obtained plasma samples from animals that underwent sham surgery (*n* = 2) or MI surgery at 24 (n = 2), and 48 hour post-surgery (*n* = 4). Using a comprehensive plasma proteomic analysis approach, each sample was prepared with one technical replicate, resulting in a total of 16 samples processed using an automated proteomics workflow [17]. The extraction of SWATH data was achieved using Spectronaut's DirectDIA [30,31].

**Fig. 6:**
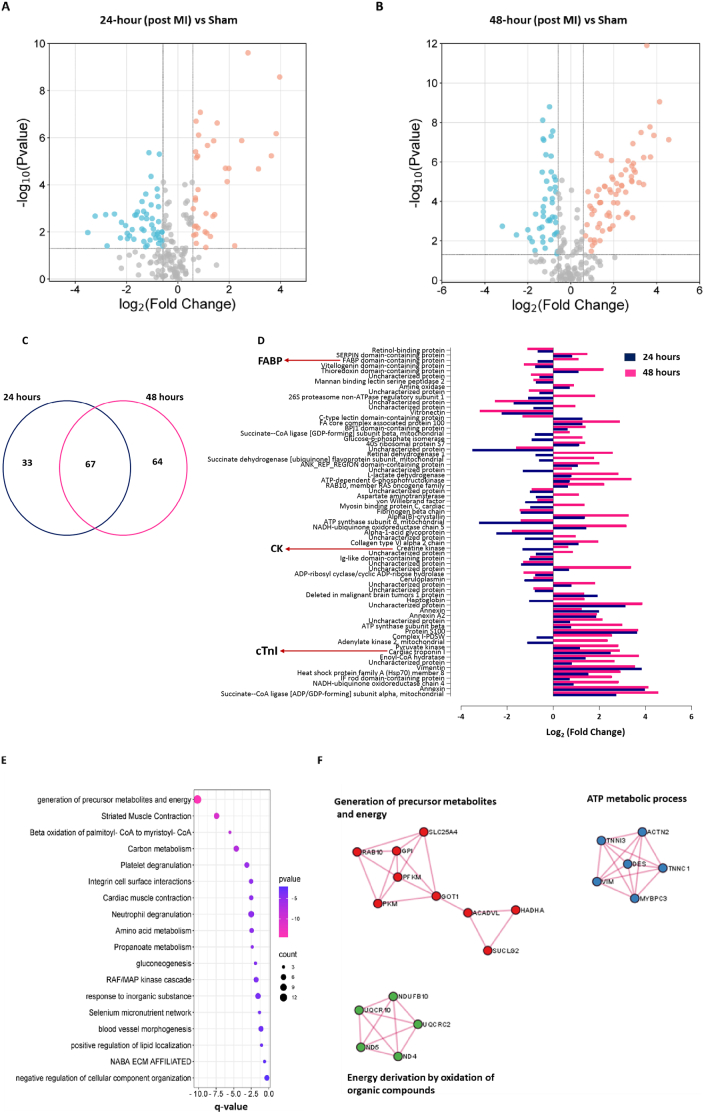
Differential expression analysis of plasma proteins in an ovine model of myocardial infarction. (A–B) Volcano plots showing the false discovery rate (FDR)-corrected *p*-values from a *t*-test comparing protein abundance at 24 h post-MI versus sham (A) and 48 h post-MI versus sham (B), plotted against log₂ fold change values (calculated from peak areas). Proteins significantly upregulated are highlighted in orange, and those significantly downregulated are highlighted in green (*p* < 0.05 after FDR correction and fold change ≥2). (C) Venn diagram illustrating differentially expressed plasma proteins at 24 h and 48 h post-surgery, highlighting the overlap between time points. The shared set includes established plasma cardiac biomarkers such as cardiac troponin I, creatine kinase, and fatty acid binding protein 3. (D) The comparison of 67 proteins at two time points revealed that the direction of changes was consistent for 51 proteins and opposite for 16 proteins at both the 24-hour and 48-hour time points. Established plasma cardiac biomarkers are highlighted using their respective gene symbols. (E) Pathway enrichment analysis of the 67 biomarker panel proteins was conducted using the Metascape pathways [32]. The colour in the figure indicates the significance of the enrichment (q-value); the more it shifts toward pink, the more significant the pathway is. The size of each cluster indicates the number of proteins in the cluster that are part of the pathway. (F) A protein-protein interaction network for the most representative molecular complexes is generated based on the top three functionally enriched terms automatically identified by Metascape [32]. (For interpretation of the references to colour in this figure legend, the reader is referred to the web version of this article.)

Following stringent quality control, direct SWATH data extraction identified 418 proteins in sheep plasma across the two time points, each supported by more than one unique peptide. Protein abundances were quantified using integrated peak areas (Supplementary File 5). Statistical analysis revealed a substantial number of differentially abundant proteins (with *P* < 0.05 after FDR correction and intensity change of more than two-fold) in the 24-hour and 48-hour samples, with 38 upregulated and 62 downregulated proteins in the former (Fig. 6A), and 78 upregulated and 53 downregulated proteins in the latter (Fig. 6B). To assess the consistency of a proteomic-based plasma biomarker panel, we focused on common proteins at both time points, identifying 67 consistent proteins, including established plasma cardiac biomarkers such as cardiac troponin I (*cTnI*), creatine kinase (*CK*), and fatty acid binding protein 3 (*FABP3*) (Fig. 6C). Importantly, among them 16 of these proteins were classified as mitochondrial proteins. Further analysis revealed that 51 proteins exhibited consistent directionality, while 16 showed opposite trends across the two time points (Fig. 6D). Additionally, we identified differentially expressed cardiac biomarker proteins specific to each time point, such as cardiac troponin T (*cTnT*) (Log_2_ Fold = 2.212, 48 h) positively associated with myocardial infarction and myoglobin (*MB*) (Log_2_ Fold = −2.8113, 24 h) was negatively associated.

Pathway enrichment analysis of the plasma biomarker panel revealed that the generation of precursor metabolites and energy, carbon metabolism, platelet degranulation, and neutrophil degranulation are consistent with the functional analysis of the ovine infarct tissue proteome (Fig. 6E) [17]. Specifically, the generation of precursor metabolites and energy, as well as carbon metabolism, was significantly associated with the integrated system of the TCA cycle and oxidative phosphorylation. Furthermore, pathways including cardiac muscle contraction, amino acid metabolism, gluconeogenesis, RAF/MAP kinase cascade, blood vessel morphogenesis, and ECM affiliation were identified as significant consequences of myocardial infarction (Fig. 6E). Protein-protein interaction network analysis showed that the resultant network proteins were associated with the generation of precursor metabolites and energy, ATP metabolic process, and energy derivation by oxidation of organic compounds implicating their involvement in cardiac energy metabolism (Fig. 6F).

### 3.6 Identification of putative plasma biomarkers

**Fig. 7:**
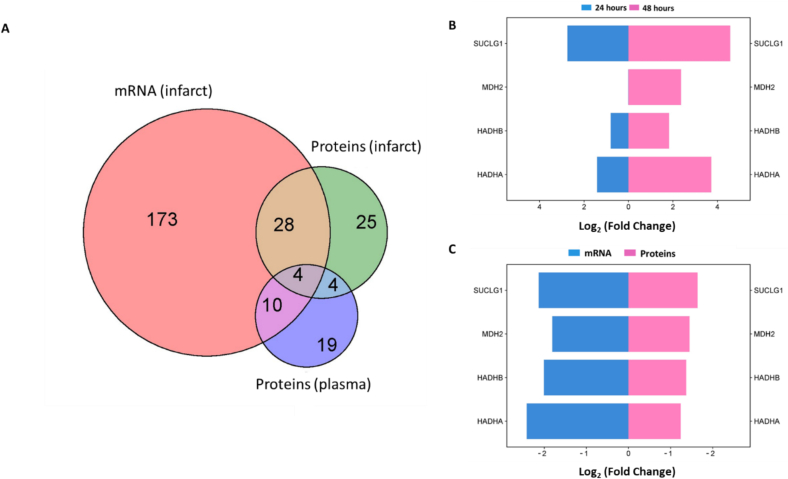
Validation of plasma biomarker panel candidates for ovine model of myocardial infarction. (A) A Venn diagram showing all mitochondrial proteins shared among datasets. Mitochondrial proteins associated with mRNA profiles (infarct vs. remote), proteomic analyses (infarct vs. remote), and plasma proteomics (24 h and 48 h) were identified using Mitocarta 3.0 [26]. Four proteins, namely *SUCLG1, MDH2, HADHB, and HADHA*, were found to be shared across all datasets. (B–C) A dual Y-axis horizontal bar plot illustrates the direction and abundance of common mitochondrial proteins in the plasma dataset at the 24-hour and 48-hour time points (B). This is presented in conjunction with the expression profiles of mRNA and their corresponding proteins from the transcriptomics and proteomics datasets, specifically infarct vs remote conditions (C).

The integration of tissue transcriptomic and proteomic analyses, focusing on differential expression patterns of mitochondrial genes and proteins in infarcted versus remote tissues [17], along with data on differentially abundant mitochondrial proteins in blood at 24- and 48-hour time points, identified four novel biomarkers: succinate-CoA ligase (*SUCLG1*)*,* malate dehydrogenase 2 (*MDH2*)*,* hydroxyacyl-CoA dehydrogenase alpha and beta subunits (*HADHB*, and *HADHA*) (Fig. 7A). *SUCLG1* and *MDH2* are key enzymes in the citric acid cycle, while *HADHB* and *HADHA* are subunits of the mitochondrial trifunctional enzyme complex involved in fatty acid β-oxidation. Both transcriptomic and proteomic data consistently revealed downregulation of these proteins in infarcted tissues (Fig. 7B), whereas their plasma concentrations were elevated, likely due to intracellular release following cellular damage.

Temporal analysis indicated that *SUCLG1, HADHB*, and *HADHA* were detectable at both 24- and 48-hour post-infarction, while *MDH2* was only detected at 48 h, suggesting temporally regulated expression (Fig. 7C). The persistent plasma presence and consistent upregulation of *SUCLG1*, *HADHB*, and *HADHA* highlight their potential as novel biomarkers for early detection and monitoring of cardiovascular events. The delayed plasma detection of *MDH2* highlights its temporal regulation in myocardial infarction. Interestingly, our approach identified a consistent upregulation of *cTnI* at both time points, whereas *cTnT* exhibited biphasic regulation initially downregulated at 24 h, followed by upregulation at 48 h. Additionally, extracellular matrix (ECM) proteins, including vitronectin (*VTN*), plasminogen (*PLG*), and fibrinogen beta chain (*FGB*), displayed significant fold changes and were strongly associated with early myocardial infarction. These findings highlight the diagnostic value of integrating mitochondrial proteins detectable in both tissue and plasma with traditional cardiac troponins, potentially enhancing the precision of cardiovascular disease diagnostics.

## 4 Discussion

Linking mitochondrial dysfunction and metabolic reprogramming in the infarcted myocardium to systemic biomarker signatures remains a significant challenge due to spatial and cellular heterogeneity within the heart and the inherently low abundance of mitochondrial proteins in circulation. To address these limitations, we applied a region-specific, integrative multi-omics strategy combining quantitative proteomics, transcriptomics, and miRNAomic analyses of infarcted and adjacent myocardial zones. Our mitochondrial protein analysis revealed a consistent downregulation of metabolic enzymes, which was corroborated at the transcriptomic level. Integrated analysis highlighted the inhibitory regulation of *PPARGC1A*, a master transcriptional coactivator of mitochondrial biogenesis and oxidative energy metabolism, implicating its suppression in the loss of mitochondrial function during MI. Notably, our SWATH-MS–based proteomics approach enabled the identification of disease-relevant proteins and perturbed molecular networks that were not only altered in cardiac tissue but also detectable in the circulation, underscoring their potential as plasma biomarkers of MI. These findings offer new opportunities for the non-invasive detection and monitoring of MI, complementing current diagnostic tools. Moreover, by enhancing the molecular resolution of cardiac tissue remodeling, our data contribute to the discovery of novel diagnostic and therapeutic targets, thereby advancing the translational relevance of large-animal models in cardiovascular research and supporting the development of precision medicine strategies.

The proteomics analysis revealed substantial heterogeneity in the abundance of mitochondrial proteins within infarct samples, suggesting markedly reduced mitochondrial energy metabolism that contributes to mitochondrial dysfunction in infarcted regions compared to neighbouring regions within the same tissue. This observation was consistent with the current understanding of energy metabolism in adult cardiomyocytes, as well as the regulation of cardiac fibrosis and extracellular matrix accumulation following infarction [13,15,16]. Furthermore, a significant reduction in overall abundance of mitochondrial proteins consistent with the infarcted region elucidates structural and functional alterations within the infarcted region, thereby reinforcing the increasing evidence implicating mitochondrial dysfunction and altered dynamics in the pathogenesis of myocardial infarction [8]. The elucidation of the precise mechanism governing alterations in mitochondrial protein levels in response to myocardial infarction-induced pathogenesis remains incomplete, causing uncertainty regarding whether oxidative stress, calcium-mediated mitochondrial damage and clearance, impaired mitochondrial biogenesis, or a convergence of these factors is the primary driver of changes in the functional regulation of the infarcted region [8,33,34]. However, such pronounced variations in mitochondrial protein mass observed in comparison to adjacent regions, particularly a clinically relevant large animal model, highlight significant spatial heterogeneity in mitochondrial distribution and function.

Upstream transcriptional regulators orchestrate complex protein regulatory networks essential for cellular adaptation and repair, yet their precise roles in myocardial infarction (MI) remain incompletely defined. Through integrative bioinformatic analyses using Ingenuity Pathway Analysis (IPA), we identified several key regulators *STAT3, IRF2, ZEB1, MYC*, and *PPARGC1A* previously implicated in MI pathophysiology [8,[35], [36], [37], [38]]. Notably, *MYC, ZEB1*, and *PPARGC1A* emerged as convergent regulators across our proteomic, transcriptomic, and miRNAomic datasets, suggesting their central involvement in the post-infarction response. Among them, *PPARGC1A* a master transcriptional coactivator critical for mitochondrial biogenesis and energy metabolism was uniquely predicted to be suppressed, consistent with the observed reduction in mitochondrial protein abundance and proteome remodeling within infarcted myocardium. Under physiological conditions, *PPARGC1A* activates genes involved in the TCA cycle, OXPHOS, and fatty acid β-oxidation to meet the heart's high aerobic energy demands. However, during ischemia-induced oxygen deprivation, the suppression of *PPARGC1A* drives a coordinated downregulation of these mitochondrial pathways, leading to impaired ATP production and a metabolic shift toward anaerobic glycolysis [[39], [40], [41]]. Concurrently, transcription factors *MYC* and *ZEB1* exacerbate mitochondrial dysfunction through both transcriptional and post-transcriptional mechanisms. *MYC* governs the expression of essential mitochondrial biogenesis genes, including *TFAM*, *POLG*, and *TOM20*, yet ischemic stress shifts metabolism toward glycolysis, promoting ROS accumulation and bioenergetic failure. In parallel, *ZEB1* represses mitochondrial fusion genes such as *Mfn2*, disrupting mitochondrial dynamics and further increasing oxidative stress [42,43]. While this metabolic reprogramming may initially represent an adaptive survival response, its sustained activation becomes maladaptive, driving energy deficits, mitochondrial impairment, and adverse cardiac remodeling. The predicted activation of *MYC* and *ZEB1*, both subject to miRNA-mediated regulation, highlights their central roles as modulators of mitochondrial metabolism and cellular plasticity following myocardial infarction [44,45] Moreover, locally produced *IL6* amplifies mitochondrial dysfunction via *STAT3*-dependent signaling, exacerbating ROS generation and impairing oxidative phosphorylation in cardiomyocytes [46]. Collectively, these intertwined regulatory perturbations orchestrate maladaptive metabolic remodeling, mitochondrial dysfunction, and structural deterioration of the infarcted myocardium.

The findings presented in this study highlight the translational potential of proteomic profiling in ovine cardiac tissues as a powerful approach for the discovery of clinically relevant biomarkers of myocardial injury. Despite the inherent limitations associated with the partial representation of the arterial proteome in peripheral blood plasma, our data reveal that specific mitochondrial metabolic proteins namely succinyl-CoA ligase subunit alpha, malate dehydrogenase, and the alpha and beta subunits of hydroxyacyl-CoA dehydrogenase are detectably released into circulation following myocardial infarction. These proteins demonstrate promise as novel indicators of cardiac injury due to their temporal expression, offering the potential to predict differential tissue damage up to 48 and 24 h prior to histological manifestation, based solely on their plasma abundance. Importantly, the early elevation of succinyl-CoA ligase and malate dehydrogenase reinforced the pivotal role of the TCA cycle in the metabolic response to acute myocardial injury. This was consistent with existing evidence emphasizing the centrality of mitochondrial dysfunction and metabolic reprogramming in the pathophysiology of cardiac ischemia [47,48]. Moreover, the detection of hydroxyacyl-CoA dehydrogenase subunits key enzymes involved in fatty acid β-oxidation further supported the hypothesis that impaired lipid metabolism is an early and detectable hallmark of heart failure progression [49,50]. These data collectively support a model in which mitochondrial metabolic dysregulation precedes overt tissue pathology and suggested that plasma-based detection of mitochondrial enzymes may offer a minimally invasive strategy for the early diagnosis and monitoring of myocardial infarction. While troponin assays are widely regarded as the gold standard for diagnosing ischemic and reperfusion-induced myocardial infarction, they primarily detect cardiac damage after significant myocardial injury has occurred [51,52]. In contrast, the identification of novel biomarkers provides a more sensitive and precise diagnostic approach, particularly in models such as the sheep, where permanent coronary ischemia offers distinct pathological insights compared to reperfusion injury. Notably, reperfusion injury precedes the rise of troponin, a well-established biomarker of myocardial injury, which typically increases in the bloodstream 2–4 h after the onset of MI. This temporal distinction is critical, as reperfusion activates additional cellular mechanisms that alter the biomarker profile [53]. These insights underscore the importance of identifying biomarkers that are responsive to early ischemic events, independent of reperfusion-mediated damage.

The incorporation of such biomarkers could enhance diagnostic specificity and sensitivity, enabling the detection of myocardial injury at stages prior to irreversible tissue damage. Moreover, their application may extend beyond ischemic heart disease. For instance, this approach holds translational potential for non-invasive detection of congenital heart defects during pregnancy. Biomarkers released from the fetal heart into the maternal circulation may serve as early indicators of cardiac anomalies, potentially preceding detection by conventional imaging modalities such as ultrasound. Future studies should focus on validating these biomarkers in the context of smaller infarcts and at earlier time points to evaluate their diagnostic sensitivity and temporal dynamics. Additionally, the mechanistic insights gained from such studies may illuminate novel therapeutic targets and contribute to a more comprehensive understanding of cardiovascular disease progression. Ultimately, these advances will support the development of more effective diagnostic and treatment strategies for myocardial infarction and other cardiovascular disorders.

### 4.1 Limitations of the study

This study was intended to identify mitochondrial metabolic remodeling and gene regulatory networks involved in the pathogenesis of myocardial infarction by analysing infarcted and adjacent myocardial regions in a preclinical ovine model, while acknowledging certain analytical limitations. The detection of variations in 62 mitochondrial proteins across three neighbouring regions within the same tissue represents a significant milestone. Despite implementing stringent statistical thresholds to minimise false positives (typically <1 %), the potential for a dynamic range of proteins and signal interference persists due to co-eluting peptides, which may obscure both positive and negative protein associations. Validation and reinforcement of our tissue proteomics findings were achieved through transcriptomics via microarray analysis of infarct tissues. In the context of undepleted plasma, the dynamic range of plasma proteins significantly impacts the detection of low-abundance or tissue leakage proteins, potentially limiting accessibility to these components in plasma samples. Nonetheless, 16 mitochondrial proteins were successfully identified at both time points. The confirmation and reinforcement of the presence of four mitochondrial proteins (*SUCLG1, MDH2, HADHB, and HADHA*) in plasma through transcriptomics and DIA-based proteomics of infarct tissues further supports the validity of the findings. It is imperative to acknowledge that this study employed a small number of replicates at each time point, indicative of a pilot study. This strategic choice allowed for the assessment of feasibility, identification of potential challenges, and refinement of the experimental design, laying the groundwork for subsequent larger-scale investigations. Further efforts are necessary to define the functional abnormalities associated with the identified mitochondrial proteins and determine the therapeutic implications of manipulating them. Despite this need for additional exploration, the data presented herein underscore the clinical utility of mitochondrial proteins as potential early biomarkers for myocardial infarction, even as their systemic underpinnings continue to be elucidated.

## 5 Conclusion

In conclusion, this study presents the most comprehensive proteomic characterization of mitochondrial metabolic remodeling in the ischemic myocardium of a clinically relevant large-animal (sheep) model of myocardial infarction to date. The proteomics approach revealed a broad array of differentially expressed proteins, pathways, and regulatory networks strongly indicative of early infarction and highlighted pronounced metabolic dysregulation relative to adjacent myocardial zones that are structurally affected or may be viable. By combining proteomic, transcriptomic, and miRNAomic analyses, we were able to capture distinct tissue phenotypes and delineate dynamic alterations in protein networks that reflect disease-specific molecular signatures. Importantly, our findings underscore the translational value of tissue proteomics for the identification of circulating biomarkers, particularly those related to mitochondrial metabolic function, detectable with temporal resolution in plasma. Together, this dataset and methods provide a robust foundation for future mechanistic and biomarker discovery studies, enhancing the utility of the sheep MI model for translational cardiovascular research.

The following are the supplementary data related to this article.

Supplementary 1 Quantified mitochondrial proteins across different heart regions. Supplementary 1

Supplementary 2 Quantified mitochondrial mRNAs in the remote region (no significant changes). Supplementary 2

Supplementary 3 Quantified extracellular matrix proteins and mRNAs across regions. Supplementary 3

Supplementary 4 Identified upstream regulators using Ingenuity Pathway Analysis software. Supplementary 4

Supplementary 5 Quantified plasma proteins at 24 h and 48 h time points. Supplementary 5

## CRediT authorship contribution statement

**Selvam Paramasivan:** Writing – original draft, Visualization, Validation, Software, Methodology, Investigation, Formal analysis, Data curation, Conceptualization. **Mitchell C. Lock:** Writing – review & editing, Methodology, Data curation, Conceptualization. **Roberto A. Barrero:** Writing – review & editing, Software, Methodology, Data curation. **Paul C. Mills:** Writing – review & editing, Supervision. **Janna L. Morrison:** Writing – review & editing, Supervision, Funding acquisition, Conceptualization. **Pawel Sadowski:** Writing – review & editing, Supervision, Investigation, Conceptualization.

## Declaration of Generative AI and AI-assisted technologies in the writing process

AI-assisted technology is not used in the preparation of this work (except checking grammar and spelling).

## Funding

The animal component of the project was funded by a Research Themes Investment Scheme grant from the 10.13039/501100001787University of South Australia. JLM was funded by an 10.13039/501100000923Australian Research Council Future Fellowship (FT170100431, Level 3).

## Declaration of competing interest

The authors declare that they have no known competing financial interests or personal relationships that could have appeared to influence the work reported in this paper.

## Data Availability

Data supporting the findings of this manuscript are available from the authors upon reasonable request.
